# PDK1- and PDK2-mediated metabolic reprogramming contributes to the TGFβ1-promoted stem-like properties in head and neck cancer

**DOI:** 10.1186/s40170-022-00300-0

**Published:** 2022-12-06

**Authors:** Wan-Hsuan Sun, Yun-Hsuan Chen, Hou-Hsuan Lee, Yu-Wen Tang, Kuang-Hui Sun

**Affiliations:** 1 Division of Head & Neck Surgery, Department of Otolaryngology, Tri-Service General Hospital and National Defense Medical Center, Taipei, 112 Taiwan, Republic of China; 2 Department of Biotechnology and Laboratory Science in Medicine, Cancer Progression Research Center, National Yang Ming Chiao Tung University, #155, Section 2, Lie-Nong Street, Taipei, 112 Taiwan, Republic of China; 3 Division of Oral & Maxillofacial Surgery, Department of Stomatology, Taichung Veterans General Hospital, Taichung, 407 Taiwan, Republic of China; 4 Department of Education and Research, Taipei City Hospital, Taipei, 112 Taiwan, Republic of China

**Keywords:** PDK1, PDK2, Cancer stem cells, TGFβ1, Head and neck cancer

## Abstract

**Background:**

Resistance to chemotherapeutic drugs is a key factor for cancer recurrence and metastases in head and neck cancer (HNC). Cancer stem cells (CSCs) in tumors have self-renewal, differentiation, and higher drug resistance capabilities, resulting in a poor prognosis for patients. In glucose metabolism, pyruvate dehydrogenase kinase (PDK) inhibits pyruvate dehydrogenase and impedes pyruvate from being metabolized into acetyl-CoA and entering the tricarboxylic acid cycle to generate energy. Studies have reported that PDK1 and PDK2 inhibition suppresses the growth, motility, and drug resistance of cancer cells. Furthermore, while TGFβ1 levels are persistently elevated in HNC patients with poor prognosis, the role of PDK isoforms in the TGFβ1-promoted progression and stem-like properties of HNC is unclear.

**Methods:**

Levels of PDK1 and PDK2 were evaluated in HNC tissue microarrays by immunohistochemistry to explore potential clinical relevance. PDK1 and PDK2 were knocked down by the lentivirus shRNA system to investigate their role in TGFβ1-promoted tumor progression in vitro.

**Results:**

We found that PDK2 levels were increased in the later stage of HNC tissues compared to constant PDK1 expression. After PDK1 and PDK2 knockdown, we discovered increased ATP production and decreased lactate production in TGFβ1-treated and untreated HNC cells. However, only PDK2 silencing significantly inhibited the clonogenic ability of HNC cells. We subsequently found that TGFβ1-promoted migration and invasion capabilities were decreased in PDK1 and PDK2 knockdown cells. The tumor spheroid-forming capability, motility, CSC genes, and multidrug-resistant genes were downregulated in PDK1 and PDK2 silencing CSCs. PDK1 and PDK2 inhibition reversed cisplatin and gemcitabine resistance of CSCs, but not paclitaxel resistance.

**Conclusion:**

The results demonstrated that the PDK1- and PDK2-mediated Warburg effect contributes to the TGFβ1-enhanced stemness properties of HNC. Therefore, PDK1 and PDK2 may serve as molecular targets for the combination therapy of HNC.

**Supplementary Information:**

The online version contains supplementary material available at 10.1186/s40170-022-00300-0.

## Background

According to the American Cancer Society, head and neck cancer (HNC) is among the top ten new cases of cancer, and more than half of patients experience relapse and metastasis after treatment [1]. Currently, surgical intervention combined with chemotherapy and radiotherapy is the main treatment for HNC. More than half of patients suffering from distant metastasis have less than a 5% survival rate after platinum-based chemotherapy [2]. Immunotherapy that includes cetuximab to target epidermal growth factor receptor (EGFR) is an alternative treatment, but the result can reduce the mortality rate by only 20% and prolong survival time 2.7 months, even when combined with platinum-based chemotherapy [3]. Studies have found that cancer stem cells (CSCs) with self-renewal and differentiation capabilities may cause cancer metastasis, relapse, and drug resistance; therefore, targeting CSCs is currently the main focus of cancer treatments [4].

The dynamic crosstalk between cancer cells and the tumor microenvironment (TME) is essential for CSC formation and tumor progression. Interactions between cancer cells and TME occur via cell-cell or cell-extracellular matrix contacts and mediators. The stromal cells of TME contain immune cells, endothelial cells, pericytes, fibroblasts, etc., and the mediators include secreted soluble factors and vesicles, such as chemokines, cytokines, growth factors, and exosomes [5]. One of the key factors is transforming growth factor-beta (TGFβ), which plays a vital role in TME to augment tumorigenesis by promoting cell transformation, angiogenesis, and CSC formation and modulating immune cells and stroma components [5, 6]. CSCs can also reprogram their metabolism to survive under adverse conditions and display different metabolic features depending on the TME [7]. Cancer cells tend to favor fermentation to rapidly obtain energy, even in aerobic conditions (aerobic glycolysis) during tumor progression. This phenomenon is known as the Warburg effect, which is more evident in CSCs [7, 8].

Previous studies have revealed that proliferative cancer cells can activate hypoxia-inducible factor to upregulate several protein expressions and functions, such as glucose transporter (GLUT) to increase glucose uptake, lactate dehydrogenase A (LDHA) to increase lactate production, and pyruvate dehydrogenase kinase (PDK) to inhibit mitochondrial oxidative phosphorylation [7, 8]. PDK in glucose metabolism inhibits pyruvate dehydrogenase complex (PDH) activity and impedes pyruvate from being metabolized into acetyl-CoA and entering the tricarboxylic acid cycle to generate energy. PDK has four isozymes: PDK1, PDK2, PDK3, and PDK4 [9]. Several studies have proven the connection between PDK and cancer progression. In renal cancer cells, inhibiting PDK reduced tumor size and angiogenesis in vivo [10]. A study of breast CSCs revealed that PDK1 activated glycolysis and was required to enhance stemness in hypoxia [11]. PDK2 and PDK3 silencing increased chemosensitivity to 5-FU and reduced tumorigenesis in vitro in colorectal cancer and glioma, respectively [12, 13]. Inhibition of PDK4 promoted lung tumorigenesis both in vitro and in vivo [14]. However, PDK4 knockdown inhibited CD133 (+) stemness properties and overcame resistance to sorafenib in hepatoma cells [15].

In HNC, PDK1 expression is upregulated and strongly associated with poor prognosis of patients [16]. The inhibition of PDK1 expression and its activity significantly blocks EGF-enhanced cell motility and lung metastasis of HNC cells [17]. Meanwhile, dichloroacetic acid (DCA, PDK inhibitor) combined with cetuximab (EGFR blocker) considerably reduces tumor growth in cetuximab-resistant HNC xenograft models [18]. PDK2 expression is associated with cisplatin resistance and DCA-reversed resistance of HNC cells in vitro and in vivo [19]. The CSCs of HNC showed a greater dependence on glycolysis for energy supply to sustain the stemness status [20]. However, the role of PDK isoforms in the CSCs of HNC remains unclear. Besides, the plasma levels of TGFβ1 are significantly increased in HNC patients [21], and TGFβ1 promotes CSC properties of HNC [22]. TGFβ modulates oxidative metabolism in hepatocellular carcinoma and TME [6, 23]. Therefore, the role of PDK isoforms in the TGFβ1-promoted stemness features of HNC requires further investigation. In this study, we found levels of PDK2 were increased in the later stage of HNC tissues. TGFβ1-induced Warburg effect and motility were reversed by PDK1 and PDK2 knockdown in HNC cells. PDK1 and PDK2 silencing downregulated the tumor sphere formation, motility, CSC genes, and multidrug-resistant genes in HNC CSCs. Moreover, PDK1 and PDK2 inhibition increased CSC sensitivity to cisplatin and gemcitabine. These data suggest that PDK1- and PDK2-mediated metabolic rewiring can contribute to TGFβ1-augmented stemness features of HNC.

## Methods

### Immunohistochemistry (IHC)

Paraffin-embedded tissue microarrays (TMA) including 70 HNC samples (20 oral cases, 5 pharynx cases, 8 nasal cases, 37 larynx cases) were purchased from Biomax (US Biomax Inc., Rockville, MD, USA). A waiver was granted by IRB of Tri-Service General Hospital. The characteristics of these HNC patients are shown in Supplementary Table 1. Primary IHC antibodies against PDK1 (1:100) and PDK2 (1:600) were obtained from Santa Cruz (Santa Cruz Biotech. Inc., Dallas, TX, USA). The TMA slides were de-paraffin, antigen retrieved, and blocked with dual endogenous enzyme block (Dako North America Inc., Carpinteria, CA, USA), and then primary antibodies were added at 4°C overnight followed by processing with EnVision+Dual Link System-HRP (DAB+) kits (Dako). After counterstaining with hematoxylin, a digital stained cell score was obtained using the Aperio ImageScope (Aperio Technologies Inc., Vista, CA, USA). The final score was the sum of the percentage of the stained area (0, 1, 2, 3, and 4) and intensity (0, 1, 2, 3) of the stained cells, as previously in another study [24].

### Cell culture, cell proliferation, and colony formation assay

The HNC SAS cell line was derived from poorly differentiated human squamous cell carcinoma of the tongue and cultured in DMEM medium with 10% FBS (Gibco BRL and Life Technologies). The HNC FaDu cell line was derived from a squamous cell carcinoma of the hypopharynx and cultured in RPMI medium with 10% FBS (Gibco BRL and Life Technologies). For cell proliferation assay, cells (3000 cells/100 μl) were seeded into a 96-well plate and treated with or without TGFβ1 (5 ng/ml, PeproTech). Cell growth was examined using MTS assay (Promega, Madison, WI, USA) at 24, 48, and 72 h. For clonogenic ability, cells (50 cells/3 ml) were seeded in a 6-well plate and treated with or without TGFβ1 (5 ng/ml) for 10 days. Colonies were fixed by methanol for 10 min and stained with 3% crystal violate for 30 min, and then we calculated the colony number.

### PDK1 and PDK2 knockdown by the lentivirus shRNA system

The pLKO.1 plasmids containing shRNA targeting human PDK1 (shPDK1#261, Clone ID TRCN0000006261; shPDK1#263, Clone ID TRCN0000006263) and PDK2 (shPDK2#314, Clone ID TRCN0000002314; shPDK2#315, Clone ID TRCN0000002315) were acquired from the National RNAi Core Facility (Academia Sinica, Taipei, Taiwan). According to the protocol of the National RNAi Core Facility, pCMVΔR8.91, pMD.G, and pLKO.1 puro plasmids with shRNA lentiviral knockdown vectors were transfected using TransIT-LT1 reagent (Mirus Bio) and packaged in HEK293T cells. HNC cells were then infected with shLuc or shPDK1 or shPDK2 lentivirus in the presence of 8 μg/ml of protamine sulfate (Merck Millipore, Billerica, MA, USA). Stable clones were selected by puromycin (2 μg/mL, Sigma-Aldrich, St Louis, MO, USA) for 14 days.

### RT-qPCR and western blotting

Cells (1 × 10^6^ cells/3 ml) were seeded in a 6-cm dish overnight. The expressions of genes were detected by RT-qPCR. Total RNA was extracted by TRIzol Reagent (Invitrogen), reversed transcribed into cDNA using the Maximal First Strand cDNA Synthesis Kit (Thermo Scientific) and then into qPCR using the Fast SYBR Green Master Mix with StepOnePlus real-time PCR system (Applied Biosystems). Primer sequences for qPCR are shown in Supplementary Table 2.

For western blotting, primary antibodies against PDK1 were obtained from Cell Signaling, while those against PDK2 and alfa-tubulin from Abcam. Cells (4×10^5^ cells/3 ml) were seeded in a 6-cm dish for 48 h. The expression levels of PDK1 and PDK2 protein were detected using HRP-conjugated secondary antibody (Jackson ImmunoResearch) and Pierce ECL substrate (Thermo Scientific).

### Pyruvate dehydrogenase (PDH) activity

Cells (4×10^5^ cells/3 ml) were seeded in a 6-cm culture dish for 48 h. The PDH activity of the cells (1×10^6^ cells) was tested by using the Pyruvate Dehydrogenase Activity Colorimetric Assay Kit (BioVision) according to the manufacturer’s instructions.

### Lactate and ATP production

Cells (4×10^5^ cells/3 ml) were pretreated with or without 5 ng/ml TGFβ1 medium for 48 h. Cells (1×10^4^ cells/200 μl) were then seeded in a 96-well plate and treated with or without 5 ng/ml TGFβ1 for 24 h, and then, the medium was changed to a serum-free medium. After incubation for 24 h, the lactate concentration in the supernatant was examined using the L-Lactate Assay kit (Abcam).

Cells (4×10^5^ cells/3 ml) were seeded in a 6-cm culture dish and treated with or without TGFβ1 (5 ng/ml) for 48 h. The ATP production of the parental (4×10^5^ cells) and spheroid cells (4×10^4^ cells) was tested by ATP Determination Kit (Invitrogen).

### Migration and invasion ability of HNC cells

HNC cells (4×10^5^/3 ml) were pretreated with or without 5 ng/ml TGFβ1 medium for 48 h. For migration activities, SAS cells (2.5× /100 μl serum-free DMEM) or FaDu cells (3×10^4^/100 μl serum-free DMEM) were seeded into the upper chamber of a transwell insert (8-μm pore, Costar), and the lower chamber contained 10% FBS DMEM. After incubation for 30 h, cells’ migration activities were determined by Liu’s stain. For invasion activities, SAS cells (3×10^4^ cells/500 μl serum-free DMEM) and FaDu cells (4×10^4^ cells/500 μl serum-free DMEM) were seeded into the upper chamber of a Matrigel Invasion Chamber (Corning), where the lower chamber contained 10% FBS DMEM. After incubation for 48 h, the invasion activities of cells were determined by Liu’s stain.

### Sphere formation ability and migration ability of spheroid cells

SAS cells (2.5×10^2^ cells/100 μl) were seeded in 96-well ultra-low attachment plates in serum-free DMEM/F12 supplemented with 20 ng/ml human EGF, 20 ng/ml human bFGF, and 1% N2 supplement (Gibco) with or without 10 ng/ml TGFβ1. Sphere numbers were counted after 10-day incubation.

For the migration ability of spheroid cells, SAS cells (1×10^4^ cells/2 ml) were seeded in 6-well ultra-low attachment plates in sphere medium with or without 10 ng/ml TGFβ1 sphere medium and cultured for 12 days. Sphere cells were incubated in complete medium with or without 10 ng/ml TGFβ1 complete medium for 48 h. Spheroid cells (2.5×10^4^ cells/100 μl 0.5% FBS DMEM) were seeded into the upper chamber of a transwell insert, while the lower chamber contained 10% FBS DMEM. After incubation for 30 h, cells’ migration activities were determined by Liu’s stain. Five fields were counted per filter in each group.

### Chemo drug sensitivity assay in SAS spheroids cells

Cisplatin, gemcitabine, and paclitaxel were obtained from Sigma-Aldrich. Parental or spheroid cells of SAS (2×10^3^ cells/100 μl spheroid medium) were seeded into 96-well white plates with concentration of different chemo drugs. After 48-h incubation, cell viability was accessed by Celltiter-Glo Luminescent Cell Viability Assay (Promega).

### Statistical analysis

SPSS software (SPSS, Inc., Chicago, IL, USA) was applied to perform all statistical analyses. Differences between two groups were assessed using Student’s *t* test. We compared protein expressions at different stages of cancer with the Mann-Whitney *U* test. The Pearson’s chi-square test was used to evaluate the association between protein expression and clinicopathologic characteristics.

## Results

### Expression levels of PDK2 were increased in advanced stages of HNC clinical tissues

**Fig. 1:**
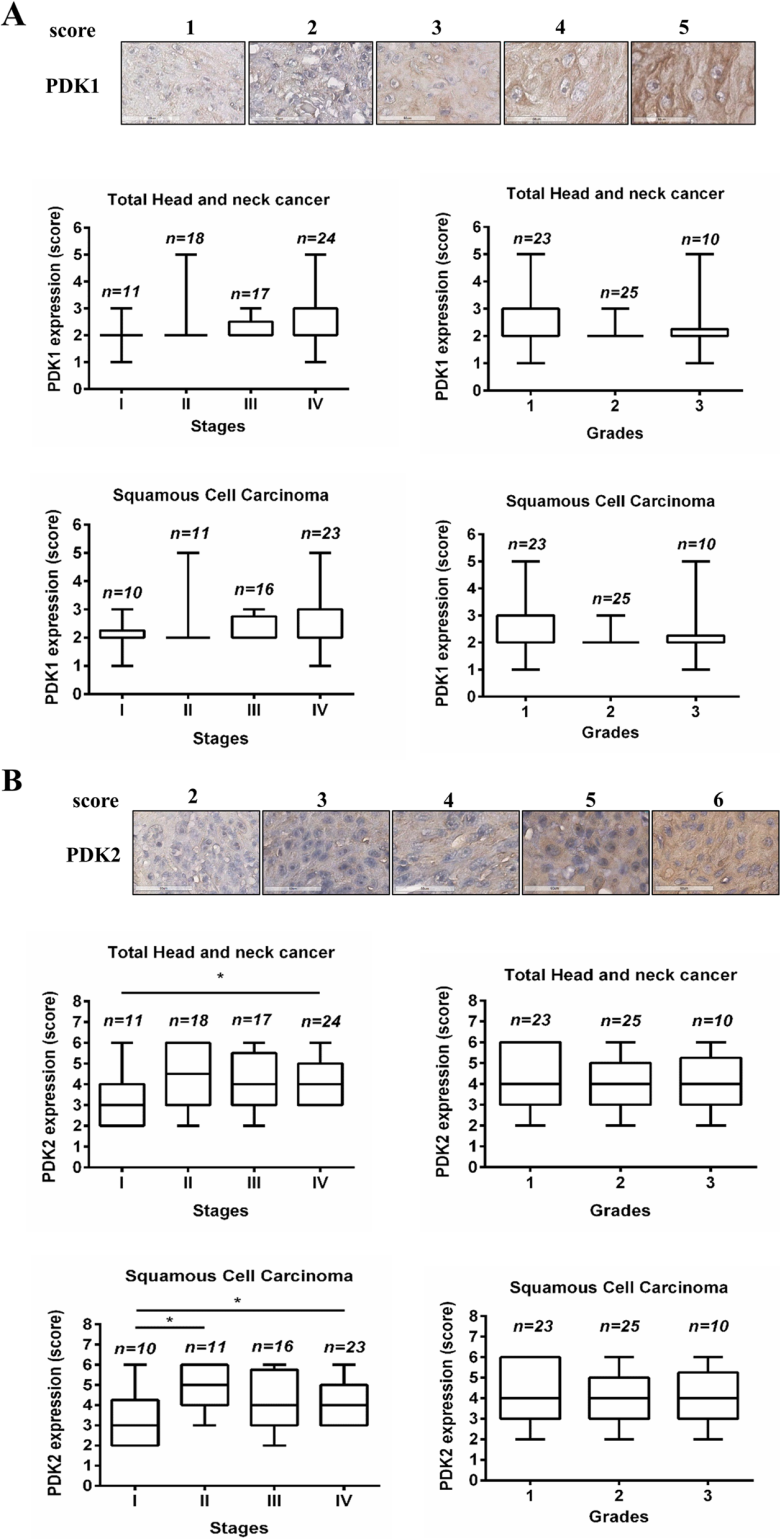
Expression levels of PDK2 were increased in higher tumor stages of HNC patients’ tissues. **A** Immunohistochemical staining was performed using anti-PDK1 on tissue microarray containing 70 cases. PDK1 levels were analyzed in various stages and grades of HNC. **B** Immunohistochemical staining was performed using anti-PDK2 on tissue microarray containing 70 cases. PDK2 levels were analyzed in various stages and grades of HNC. *p < 0.05

First, the expression levels of PDK1 and PDK2 were evaluated in HNC tissue microarrays by IHC to explore potential clinical relevance. No association between PDK1 expression levels with sex, age, T stage, node involvement (N), or metastasis (M) was noted, as shown in Table 1. However, PDK1 expression levels differed significantly between the larynx and oral cavity. The higher percentage (8/20, 40%) of PDK1 high levels was found in the oral cavity compared with the larynx (6/37, 16.2%). As shown in Fig. 1A, expression levels of PDK1 had no significant difference in the four stages or three grades of HNC and squamous cell carcinoma (HNSCC).

**Table 1:** Associations of PDK1 and PDK2 expression with clinical features of HNC patients

	No. of HNC	PDK1^a^			PDK2^b^		
		Low	High	*P*	Low	High	*P*
Sex							
F	14	11	3	0.887	7	7	0.266
M	56	43	13		19	37	
Stage							
I	11	9	2	0.688	7	4	0.048*
II+III+IV	59	45	14		19	40	
Age (years)							
<55	37	28	9	0.757	19	18	0.009*
≧55	33	26	7		7	26	
T							
T1+T2	37	29	8	0.794	15	22	0.533
T3+T4	33	25	8		11	22	
N							
N0	51	40	11	0.674	19	32	0.975
N1+N2	19	14	5		7	12	
M							
M0	67	51	16	0.430	24	43	0.681
M1	2	2	0		1	1	
Grade							
1	23	15	8	0.298	6	17	0.579
2	25	21	4		10	15	
3	10	8	2		3	7	
Location							
Larynx	37	31	6	0.046*	12	25	0.319
Oral cavity	20	12	8		4	16	

^a^Low score = 1~2. High score= 3~5

^b^Low score = 1~3. High score= 4~6

*P<0.05

Although we observed no association between PDK2 levels and sex, TNM stage, histological grade, or primary location in Table 1, high PDK2 levels were significantly associated with older age (≧55) and advanced stages. Furthermore, Fig. 1B shows higher levels of PDK2 in stages II, III, and IV of HNC and HNSCC, while all three grades also demonstrated high PDK2 levels. Therefore, the expression levels of PDK2 had more potential clinical relevance than PDK1.

### PDK1 and PDK2 silencing modulated TGFβ1-promoted Warburg effects in HNC cells

**Fig. 2:**
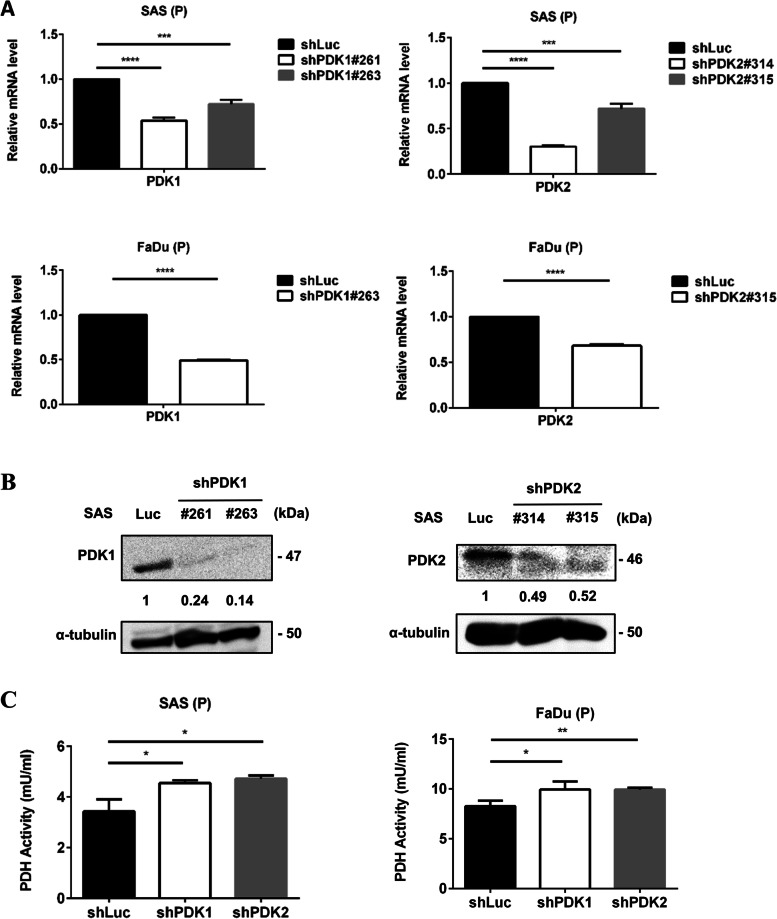
PDK1 and PDK2 knockdown were confirmed in HNC cells. **A** SAS and FaDu shLuc, shPDK1, and shPDK2 cells (1×10^6^ cells/3 ml) were seeded in a 6-cm dish overnight. PDK1 and PDK2 were detected by RT-qPCR. **B** SAS shLuc, shPDK1, and shPDK2 cells (4×10^5^ cells/3 ml) were seeded in a 6-cm dish for 48 h. The expression levels of PDK1 (left panel) and PDK2 (right panel) protein were detected by western blotting. **C** SAS shLuc, shPDK1#261, shPDK2#315 cells, FaDu shLuc, shPDK1#263, shPDK2#315 (4×10^5^ cells/3 ml) were seeded in a 6-cm culture dish for 48 h. We tested the PDH activity of the cells (1×10^6^ cells). *p < 0.05; **p < 0.01; ***p < 0.005; ****p < 0.0001

**Fig. 3:**
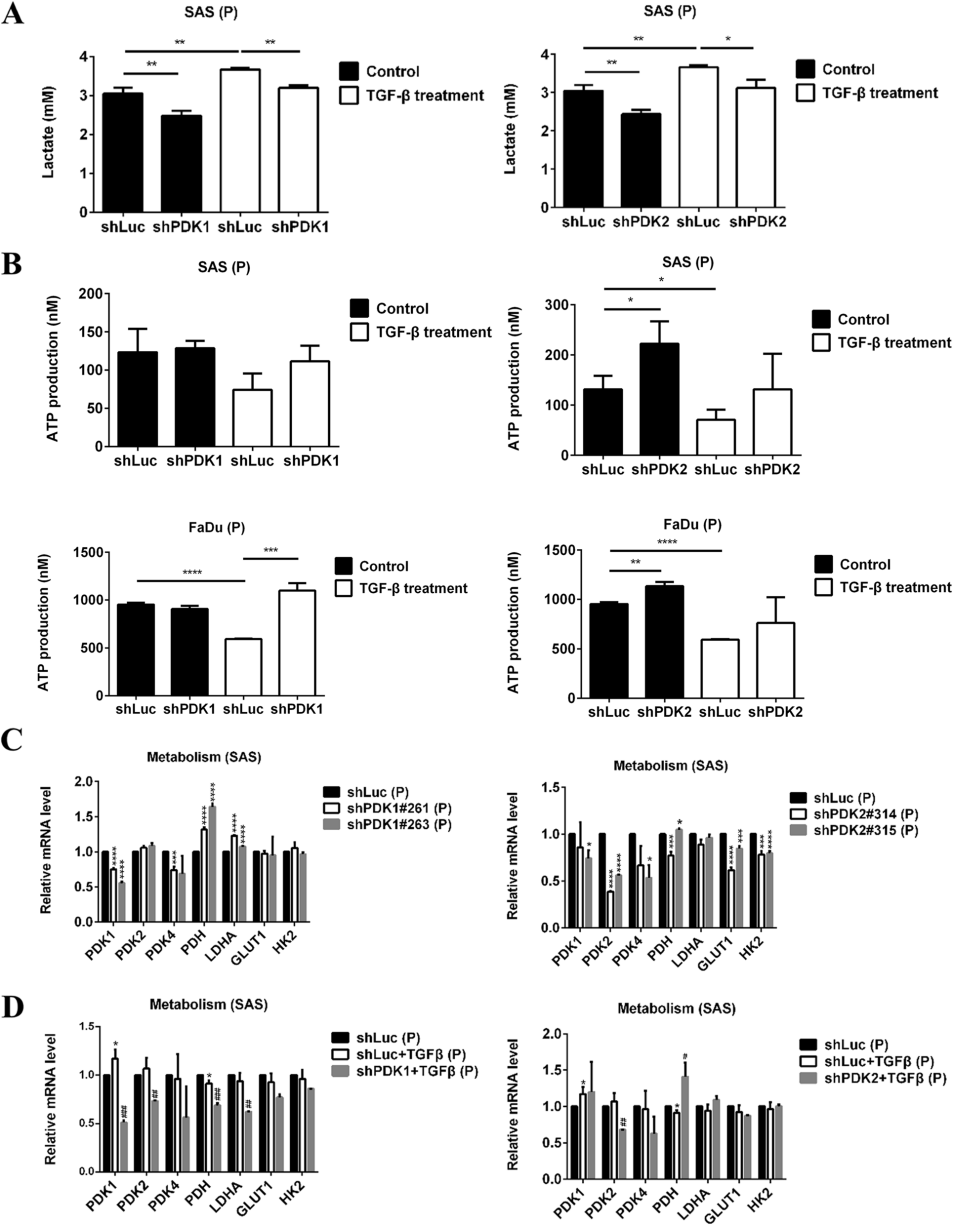
PDK1 and PDK2 silencing modulated TGF-β1-promoted Warburg effects in HNC cells. **A** SAS shLuc, shPDK1#261, and shPDK2#315 cells (4×10^5^ cells/3 ml) were pretreated with or without 5 ng/ml TGF-β1 medium for 48 h. Cells (1×10^4^ cells/200 μl) were then seeded in a 96-well plate with or without TGF-β1 for 24 h, and then the medium was changed to a serum-free medium. After incubation for 24 h, lactate concentration in the supernatant was examined using the L-Lactate Assay kit. **B** SAS shLuc, shPDK1#261, and shPDK2#315 cells and FaDu shLuc, shPDK1#263, and shPDK2#315 (4×10^5^ cells/3 ml) treated with or without TGF-β1 were seeded in a 6-cm culture dish for 48 h. We measured the ATP production of the cells (4×10^5^ cells). **C** SAS shLuc, shPDK1, and shPDK2 cells (1×10^6^ cells/3 ml) were seeded in a 6-cm dish overnight. Metabolism gene expressions were detected by RT-qPCR. **D** SAS shLuc, shPDK1#261, and shPDK2#315 cells (1×10^6^ cells/3 ml) treated with or without TGF-β1 were seeded in a 6-cm dish overnight. Metabolism gene expressions were detected by RT-qPCR. *p < 0.05; **p < 0.01; ***p < 0.005; ****p < 0.0001

To explore the role of PDK in the progression of HNC, we used a lentivirus-mediated shRNA system to knockdown PDK1 and PDK2 in HNC cells. Both PDK1 and PDK2 knockdown were confirmed in SAS and FaDu cells by using RT-qPCR and western blot. RNA expression levels of PDK1 were silenced in about 30% (shPDK1#263) and 50% (shPDK1#261), while PDK2 levels were downregulated in about 30% (shPDK2#315) and 70% (shPDK2#314) in SAS cells (Fig. 2A). In protein levels (Fig. 2B), PDK1 was decreased in about 80% and PDK2 was inhibited 50% by both clones. Furthermore, PDK could inactivate PDH, and thus the activity of PDH was examined in shPDK HNC cells. Both PDK1 and PDK2 silencing significantly upregulated the activity of PDH (Fig. 2C) and decreased lactate production (Fig. 3A) in HNC cells. However, increased ATP production was only found in shPDK2 cells (Fig. 3B).

Subsequently, we added TGFβ1 to HNC cells and found that TGFβ1 treatment increased lactate production and decreased ATP production, while the Warburg effect became more apparent in HNC cells (Fig. 3A, B). PDK1 and PDK2 knockdown in such environments decreased lactate production, increased ATP production, and suppressed the Warburg effect enhanced by TGFβ1 induction (Fig. 3A, B). Therefore, PDK1 and PDK2 silencing could modulate TGFβ1-enhanced Warburg effects in HNC cells.

To examine whether PDK silencing influenced other metabolic gene expressions, SAS cells were treated with or without TGFβ1. Suppressing PDK1 increased PDH and LDHA expression, while silencing PDK2 decreased GLUT1 and hexokinase 2 (HK2) expression (Fig. 3C). Under TGFβ1 treatment, PDK1 knockdown decreased PDH and LDHA expression, while PDK2 silencing increased PDH expression (Fig. 3D).

### PDK2 knockdown suppressed cell proliferation and clonogenic ability of HNC cells

**Fig. 4:**
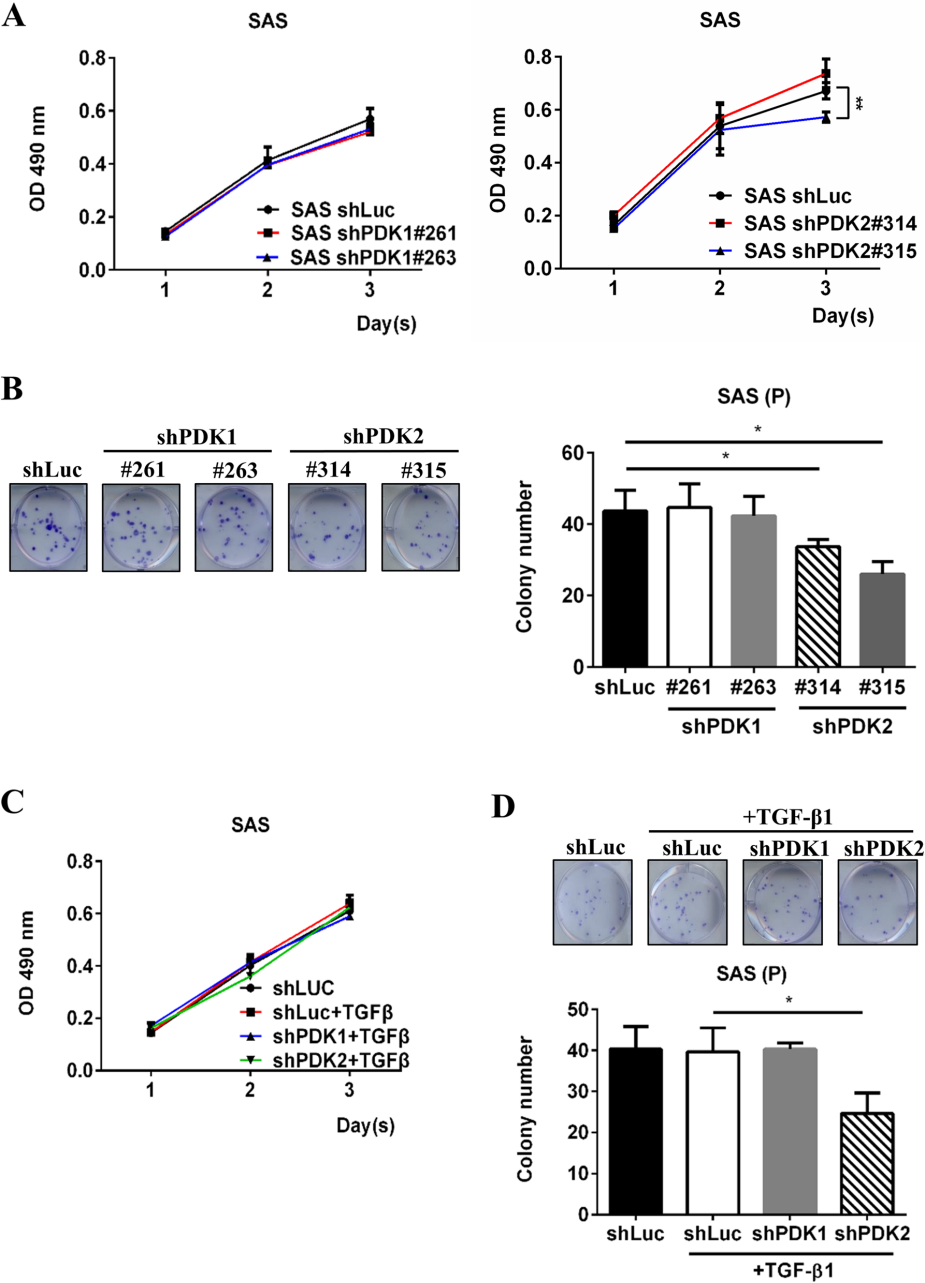
PDK2 knockdown suppressed cell proliferation and clonogenic ability of HNC cells. **A** SAS shLuc, shPDK1, and shPDK2 cells (3000 cells/100 μl) were seeded in a 96-well plate. Cell viability was examined by MTS assay on a different day. **B** SAS shLuc, shPDK1, and shPDK2 cells (50 cells/3 ml) were seeded in a 6-well plate for 10 days. Clonogenic ability was determined by colony number. **C** SAS shLuc, shPDK1#261, and shPDK2#315 cells (3000 cells/100 μl) were seeded in a 96-well plate with or without 5 ng/ml TGF-β1. Cell viability was examined by MTS assay. **D** SAS shLuc, shPDK1#261, and shPDK2#315 cells (50 cells/3 ml) were seeded in a 6-well plate with or without TGF-β1 for 10 days, and the colony number was calculated. *p < 0.05; **p < 0.01

Next, we investigated the effects of PDK knockdown on cell growth ability. As shown in Fig. 4A, suppressing PDK1 did not affect the 3-day growth of SAS cells. However, PDK2 silencing decreased short-term cell proliferation. Moreover, shPDK2 downregulated the clonogenic ability of cells (Fig. 4B). Under TGFβ1 treatment, both PDK1 and PDK2 knockdown did not affect 3-day cell growth (Fig. 4C). In contrast to PDK1 suppression, silencing PDK2 decreased the clonogenic activity of SAS cells under TGFβ1 induction (Fig. 4D). Therefore, PDK2 knockdown influenced the colony formation ability both with and without TGFβ1 treatment.

### PDK1 and PDK2 knockdown decreased TGFβ1-promoted motility of HNC cells

**Fig. 5:**
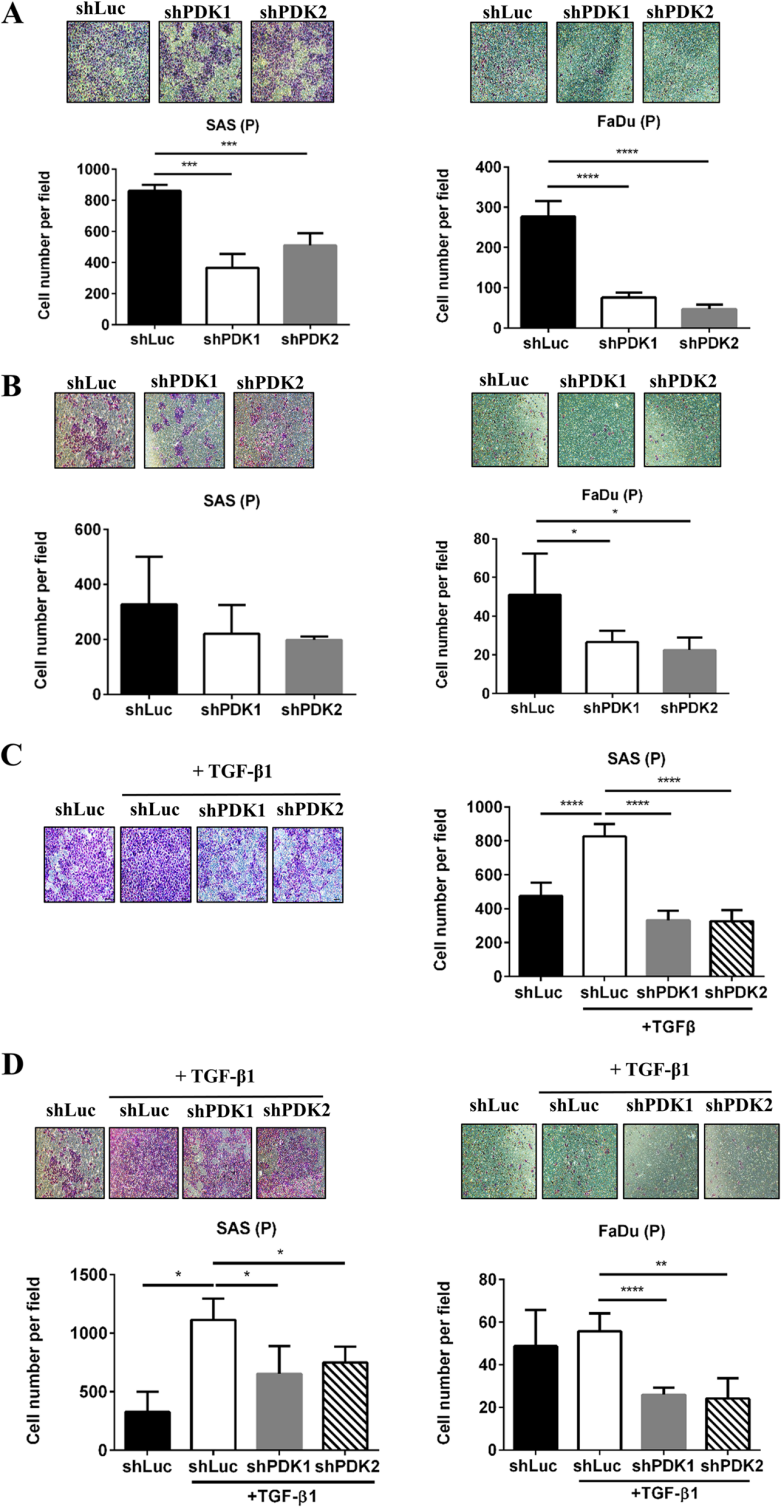
PDK1 and PDK2 knockdown decreased TGF-β1-promoted migration and invasion ability of HNC cells. **A** Transwell migration activities of SAS shLuc, shPDK1#261, and shPDK2#315 cells (2.5×10^4^/100 μl serum-free DMEM) and FaDu shLuc, shPDK1#263, and shPDK2#315 cells (3×10^4^/100 μl serum-free DMEM) were determined after 30 h. **B** Transwell invasion activities of SAS shLuc, shPDK1#261, and shPDK2#315 cells (3×10^4^ cells/500 μl serum-free DMEM) and FaDu shLuc, shPDK1#263, and shPDK2#315 cells (4×10^4^ cells/500 μl serum-free DMEM) were determined after 48 h. **C** SAS shLuc, shPDK1#261, and shPDK2#315 cells (4×10^5^/3 ml) were pretreated with 5 ng/ml TGF-β1 medium for 48 h. Migration activities of these cells were determined as described in **A**. **D** SAS and FaDu cells (4×10^5^/3 ml) were pretreated with TGF-β1 medium for 48 h. Invasion activities of these cells were determined as described in **B**. *p < 0.05; **p < 0.01; ***p < 0.005; ****p < 0.0001

We subsequently investigated the role of PDK in the mobility of HNC cells. We found that PDK1 and PDK2 silencing suppressed both the migration (Fig. 5A) and invasion (Fig. 5B) abilities of HNC cells. In an environment containing TGFβ1, the migration (Fig. 5C) and invasive (Fig. 5D) capabilities of SAS cells were enhanced. Inhibiting PDK1 and PDK2 in such environments impeded the migration and invasive abilities of HNC cells (Fig. 5C, D).

### PDK1 and PDK2 silencing downregulated sphere formation ability, stemness gene, and multidrug resistance gene expression in HNC cells

**Fig. 6:**
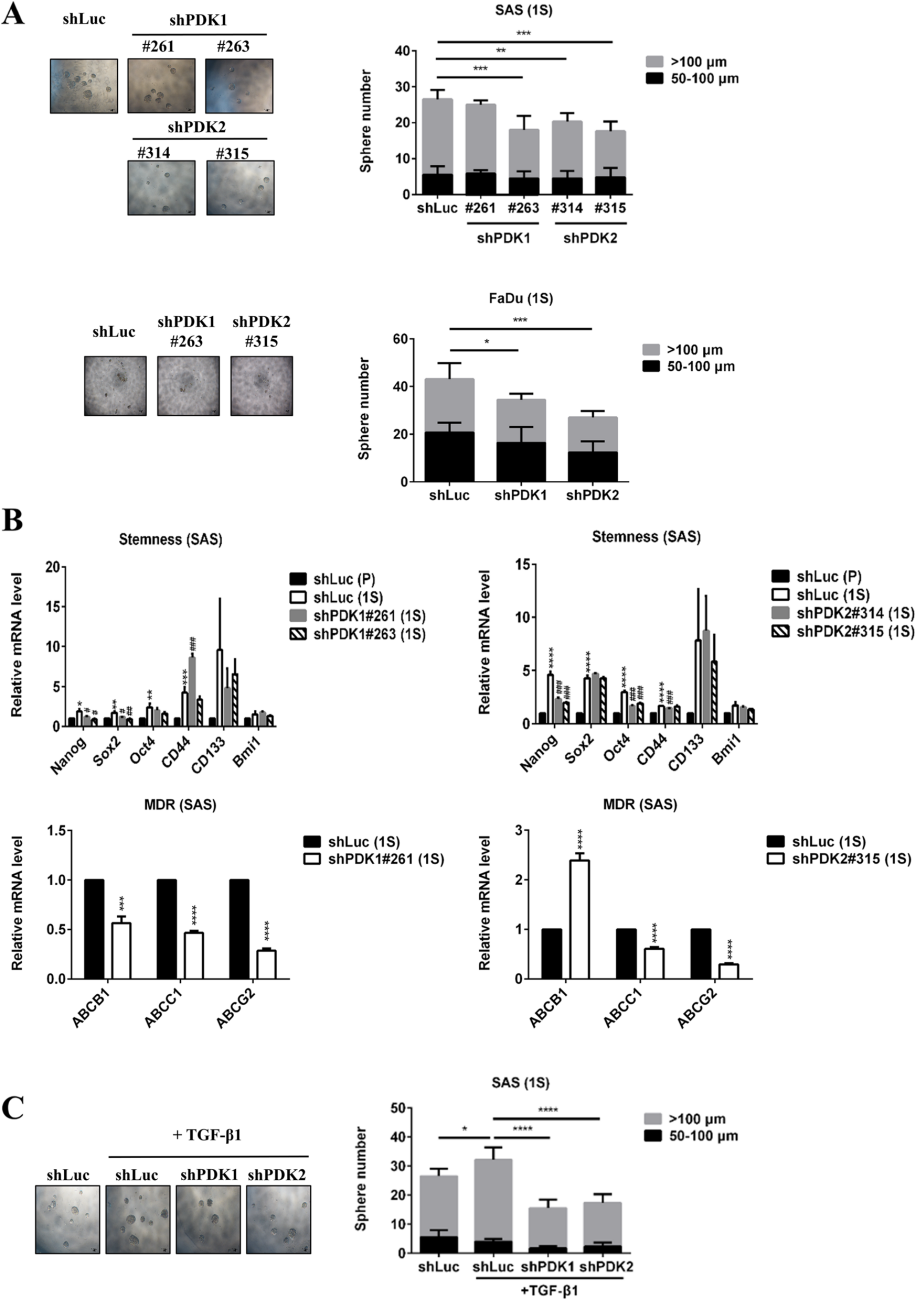
PDK1 and PDK2 silencing downregulated sphere formation ability, stemness gene, and multidrug resistance gene expression in HNC cells. **A** SAS and FaDu cells (2.5×10^2^ cells/100 μl) were seeded in 96-well ultra-low attachment plates in sphere medium. Sphere numbers were counted after 10-day incubation. **B** SAS shLuc parental cells (1×10^6^ cells/3 ml) were seeded in a 6-cm dish with sphere medium overnight. SAS cells (1×10^4^ cells/2 ml) were seeded in 6-well ultra-low attachment plates in sphere medium for 12 days. Expression of stemness genes and multidrug resistance genes (MDR) were detected in parental cells (P) and sphere cells (1S) by RT-qPCR. **C** SAS shLuc, shPDK1#261, and shPDK2#315 cells (2.5×10^2^ cells/100 μl) were seeded in 96-well ultra-low attachment plates in sphere medium with or without 10 ng/ml TGF-β1. Sphere numbers were counted after 10-day incubation. shLuc (1S) compared with shLuc (P): *p < 0.05; **p < 0.01; ***p < 0.005; ****p < 0.0001. shPDK (1S) compared with shLuc (1S): ^#^*p* < 0.05; ^##^*p* < 0.01; ^###^*p* < 0.005; ^####^*p* < 0.0001

Cancer stem cells (CSCs) have self-renewal capability and higher drug resistance, resulting in poor prognosis of patients. Suppressing PDK1 has been demonstrated to inhibit hypoxia-promoted mammosphere formation [11]. Therefore, we examined the effects of PDK knockdown on CSC formation and chemo-drug sensitivity. PDK1 and PDK2 silencing could reduce the tumor sphere formation of HNC cells (Fig. 6A). Furthermore, shPDK1 downregulated the expressions of stemness genes (Nanog, Sox2) and multidrug resistance genes (ABCB1, ABCC1, ABCG2) (Fig. 6B). PDK2 knockdown reduced the expressions of stemness genes (Nanog, Oct4) and multidrug resistance genes (ABCC1, ABCG2). In an environment containing TGFβ1, the number of tumor spheroids formed was increased (Fig. 6C). Inhibiting PDK1 and PDK2 in such environments reduced the formation of tumor spheroids.

### PDK1 and PDK2 knockdown inhibited migration ability and reversed cisplatin and gemcitabine resistance in SAS spheroids cells

**Fig. 7:**
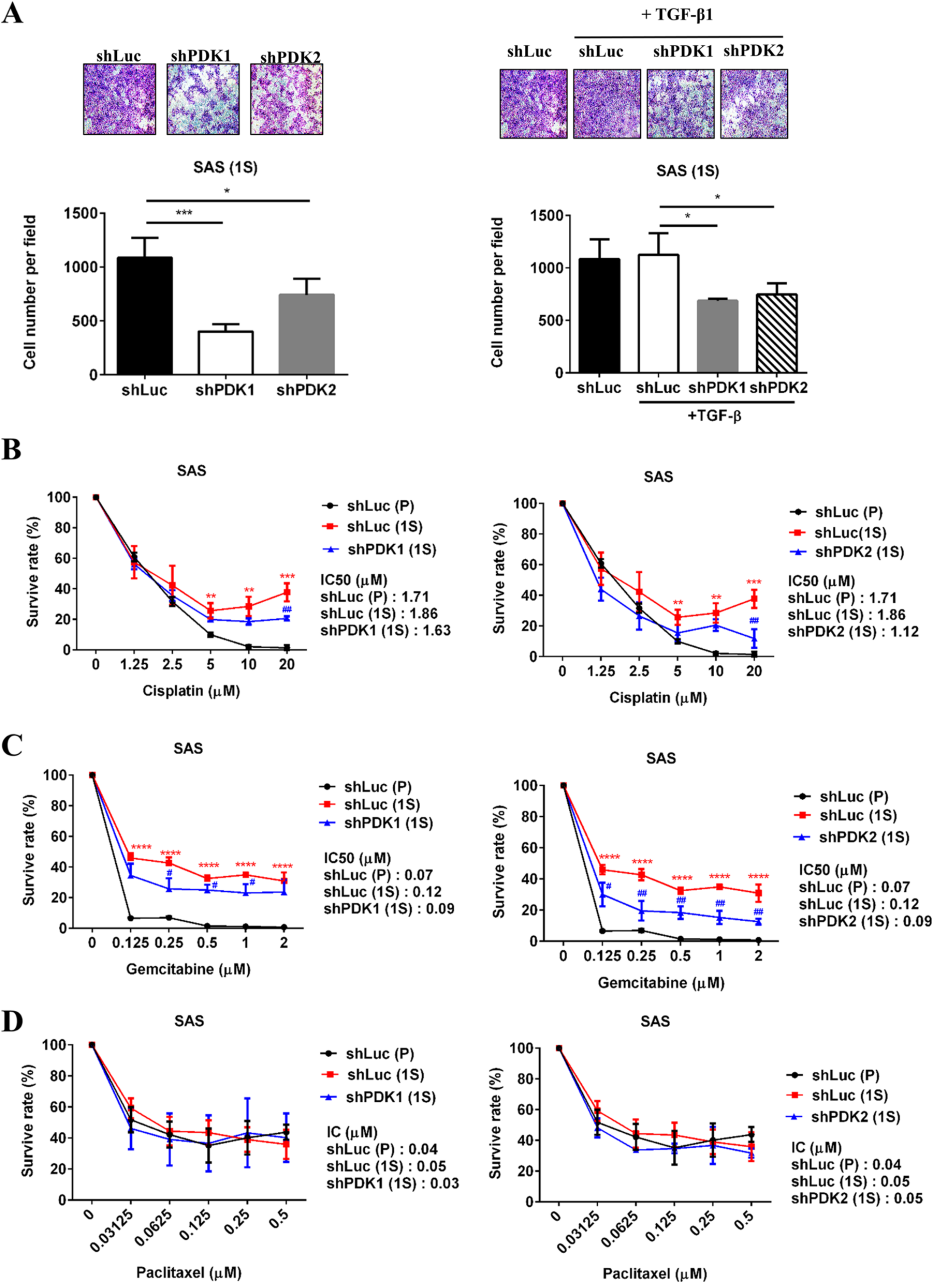
PDK1 and PDK2 knockdown inhibited migration ability and reversed cisplatin and gemcitabine resistance in SAS spheroids cells. **A** SAS shLuc, shPDK1#261, and shPDK2#315 cells (1×10^4^ cells/2 ml) were seeded in 6-well ultra-low attachment plates in sphere medium with or without 10 ng/ml TGF-β1 sphere medium and cultured for 12 days. Migration activities of the spheroid cells (2.5×10^4^ cells/100 μl 0.5% FBS DMEM) were determined after 30 h. Five fields were counted per filter in each group. **B–D** Parental (P) or spheroid (1S) cells of SAS shLuc, shPDK1#261, and shPDK2#315 cells (2×10^3^ cells/100 μl) were seeded in 96-well white plates with different chemo drugs. Celltiter-Glo Luminescent Cell Viability Assay was accessed after 48-h incubation. shLuc (1S) compared with shLuc (P): *p < 0.05; **p < 0.01; ***p < 0.005; ****p < 0.0001. shPDK (1S) compared with shLuc (1S): ^#^*p* < 0.05; ^##^*p* < 0.01

Next, we investigated the effects of PDK knockdown on the functions of CSCs. As shown in Fig. 7A, suppressing PDK1 and PDK2 reduced the motility of SAS CSCs treated with or without TGFβ1. In addition, CSCs significantly increased chemoresistance compared with parental cells, while PDK1 and PDK2 silencing reversed cisplatin (Fig. 7B) and gemcitabine (Fig. 7C) resistance of CSCs. However, CSCs did not increase chemoresistance compared with parental cells, and shPDK had no effect on the paclitaxel sensitivity of CSCs (Fig. 7D).

## Discussion

CSCs show flexible metabolic phenotypes depending on their tissue origin and TME. Mitochondrial oxidative phosphorylation is the main source of energy production for glioma and pancreatic CSCs. In contrast, breast and osteosarcoma CSCs mainly use aerobic glycolysis for energy and survival [7]. In HNC, radioresistant CSCs predominately depend on glycolytic metabolism for exhibiting stemness features [20]. Some such crucial factors as oxygen content, nutrients, and TME influence the metabolic features of CSCs. TGFβ secreted by tumors, CSCs, and stroma cells can shape the metabolism of cells within TME for angiogenesis, immune evasion, and epithelial-mesenchymal transitions (EMT) of tumor cells [4–6]. In the present study, we demonstrated for the first time that both PDK1- and PDK2-mediated aerobic glycolysis contribute to the TGFβ1-promoted stemness properties of HNC.

PDKs play a pivotal role in cancer progression by phosphorylating PDH to inhibit enzyme activity and result in a glycolytic shift [9]. Four PDK isoforms have variable tissue distribution, PDH binding affinity, and phosphorylation site-specificity. PDK3 has the highest binding affinity, then PDK1 and PDK2 have intermediate affinity, and PDK4 has the lowest affinity. Only PDK1 phosphorylates all three serine sites of PDH [9]. PDK1, PDK2, and PDK3 play a role of tumor promoters [10–13, 16–19], while PDK4 plays a different role in tumor aggressiveness depending on the cancer origin [14, 15]. In particular, PDK1 increases significantly in breast CSCs under hypoxia, and the inhibition of PDK1 may reduce the formation of mammospheres and the ability of tumor growth in vivo [11]. HNC CSCs and tumor cells prefer using aerobic glycolysis to obtain instant energy and macromolecule precursors for sustaining their indefinite proliferation [7, 20]. In this study, in addition to PDK1, we further found that PDK2 silencing reduced the stemness features of HNC CSCs, including tumor sphere formation ability, motility, CSC genes, and multidrug-resistant genes (ABC). Furthermore, inhibition of PDK1 and PDK2 overcame cisplatin and gemcitabine resistance of CSCs, but not paclitaxel resistance (Fig. 7).

The underlying mechanisms for chemodrug resistance of cancers include general and drug-specific mechanisms such as drug-target mutations, increased DNA repair processes, elevated apoptosis threshold, ATP-binding cassette transporters (ABC) activation, altered cancer metabolism, and CSC formation [25]. Cisplatin binds to and crosslinks with nuclear DNA to form DNA adducts, and gemcitabine is a nucleoside analog that prevents chain elongation following interference from DNA synthesis. One previous study revealed that PDK1 silencing induces apoptosis and overcomes cisplatin resistance in ovarian cancer-resistant cells by downregulating the EGFR activation pathway [26]. DCA, a structural pyruvate analog and PDK inhibitor, increases mitochondrial ROS production, then activation of p53 and the downstream proapoptotic pathways, to elevate chemosensitivity in cisplatin-resistant HNC cells both in vitro and in vivo [19]. Furthermore, paclitaxel stabilizes the microtubule structure by disrupting the dynamic equilibrium between soluble tubulin dimers and their polymers to induce cell death and apoptosis of cancer cells. In lung cancer, PDK inhibition reduces glycolysis and restores sensitivity of chemo-resistant cells to paclitaxel [27, 28]. DCA induces citrate accumulation and ABCB1 inactivation to reverse resistance in paclitaxel-resistant A549 cells [27]. PDK2 knockdown reduced expression levels of c-Myc and HIF-1α to increase chemosensitivity in paclitaxel-resistant A549 cells [28]. While PDK1 and PDK2 silencing increased sensitivity of HNC CSCs to cisplatin and gemcitabine, it did not do the same with paclitaxel (Fig. 7). Further research is needed to identify potential pathway-specific mechanisms for PDK-promoted chemoresistance in HNC CSCs.

Although PDK1 and PDK2 have similar biological functions in the chemoresistance and tumor progression of HNC, we found that only PDK2 was significantly elevated in the advanced stage of HNC (Fig. 1B) and that PDK2 silencing predominantly inhibited the proliferation and colony formation abilities of HNC cells (Fig. 4). PDK1 is abundantly distributed in the cardiac muscle, pancreatic islets, and skeletal muscle. PDK2 is commonly expressed in the heart, kidney, and liver [9]. Nevertheless, PDK2 is the only PDK enzyme found to be a p53 downstream target. Wild-type P53 downregulates PDK2 expression and its activity and thus promotes the oxidative phosphorylation pathway in cancer cells [9, 29]. Mutations of the *P53* tumor suppressor gene are the most common changes in HNC. *P53* mutations are associated with poor prognosis and radio- and chemo-resistance in HNC patients [30]. Moreover, p53 is commonly mutated in HPV(−) HNC cells. In HPV (+) HNC patients, p53 can be bound and inactivated by the HPV E6 protein [29, 30]. SAS and FaDu cell lines used in this study are HPV(−) HNC cells with p53 mutation. Therefore, elevated PDK2 levels may have more prominent effects in the cancer aggressiveness of HNC.

Regarding the downstream mechanisms of PDK1, Wang et al. showed that PDK1-depletion induced lung cancer cell apoptosis via suppressing the Hippo-YAP/IRS2 signaling pathway [31]. Zhang et al. found that PDK1 overexpression promoted cisplatin resistance via EGFR phosphorylation and activating its kinase activity [26]. Siu et al. demonstrated that PDK1 drives ovarian cancer tumorigenesis, metastasis, and angiogenesis via JNK/IL-8 signaling [32]. Regarding the downstream mechanisms of PDK2, Sun et al. showed that PDK2 knockdown sensitizes lung cancer cells to paclitaxel via inhibiting c-Myc and HIF-1α expression and suppresses the glucose uptake via downregulating glucose transporter-1 (GLUT1) expression [28]. DCA, a structural analog of pyruvate and served as a PDK kinase inhibitor, abolished PDK2-mediated PDH-E1α phosphorylation and increased p53 phosphorylation followed by inducing cell death and cell cycle arrest, sensitizing cisplatin-resistant HNC cells to cisplatin [19]. PDK2 transcriptionally regulated CNNM3 expression in lung adenocarcinoma and conferred the cisplatin resistance [33]. PDK2, which was hindered by microRNA-422a, fueled cell cycle process via inducing phosphorylation of RB [34]. These studies not only provided the insights into the downstream mechanisms of PDK1/2-mediated cancer progression but also demonstrated the diversity of the signaling dependency of PDK1- and PDK2-mediated biological functions.

## Conclusion

Persistently increased plasma levels of TGFβ1 are associated with poor prognosis of HNC patients after chemoradiotherapy [21]. TGFβ1 is a crucial metabolic driver in tumor-adjusting metabolic pathways for neoplastic metastasis and CSC formation in the TME [6]. In this study, we discovered that PDK1 and PDK2 are metabolic checkpoints for tumor progression and CSC features under TGFβ1 modulation within the TME of HNC. As a result, PDK1 and PDK2 represent potentially useful theranostics and molecular targets for the combinatory therapy of HNC [9].

## Supplementary Information

**Additional file 1: Supplementary Table 1**. Clinicopathologic characteristics of patients with head and neck cancer included in this study. **Supplementary Table 2.** Primer sequences used in RT-qPCR

**Additional file 2:** Original, full-length blot images of Figure 2 B

## Data Availability

Not applicable
