# Supplementary material for: PDK1- and PDK2-mediated metabolic reprogramming contributes to the TGFβ1-promoted stem-like properties in head and neck cancer
Source: Cancer Metab. 2022 Dec 6;10:23. doi: 10.1186/s40170-022-00300-0 (PMC9727917; doi:10.1186/s40170-022-00300-0)
Supplement: Supplementary file 1 — Additional file 1: Supplementary Table 1. Clinicopathologic characteristics of patients with head and neck cancer included in this study. Supplementary Table 2. Primer sequences used in RT-qPCR [file 40170_2022_300_MOESM1_ESM.docx]

| Characteristics | No. | Characteristics | No. |
| --- | --- | --- | --- |
| Sex |  | N classification |  |
| Female | 14 | N0 | 51 |
| Male | 56 | N1 | 12 |
| Stage |  | N2 | 7 |
| I | 11 | M classification |  |
| II | 18 | M0 | 68 |
| III | 17 | M1 | 2 |
| IV | 24 | Grade |  |
| Age (years) |  | 1 | 23 |
| ＜55 | 37 | 2 | 25 |
| ≧55 | 33 | 3 | 10 |
| T classification |  | Anatomic site |  |
| T1 | 13 | Pharynx | 5 |
| T2 | 24 | Nasal cavity | 8 |
| T3 | 13 | Oral cavity | 20 |
| T4 | 20 | Larynx | 37 |

**Supplementary Table 1**. Clinicopathologic characteristics of patients with head and neck cancer included in this study

**Supplementary Table 2.** Primer sequences used in RT-qPCR

| **Genes** | **Forward primer/Reverse primer** |
| --- | --- |
| PDK1 | 5’-CACCATGCCAACAGAGGTGTT-3’  5’-CCTCATTACCCAGCGTGACA-3’ |
| PDK2 | 5’-TACGTCCCCTCCCACCTCTA-3’  5’-GCCCTCATGGCATTCTTGA-3’ |
| PDK4 | 5’-CCTTTGGCTGGTTTTGGTTA-3’  5’-CCTGCTTGGGATACACCAGT-3’ |
| PDH | 5’-GGCGCAAGTTGGTGATCCTA-3’  5’-CTCCAGAAAATGCCACTCGAA-3’ |
| LDHA | 5’-AGCGGTTGCAATCTGGATTC-3’  5’-GGTGAACTCCCAGCCTTTCC-3’ |
| GLUT1 | 5’-TCACTGTGCTCCTGGTTCTG-3’  5’-CCTGTGCTCCTGAGAGATCC-3’ |
| HK2 | 5’-CAAAGTGACAGTGGGTGTGG-3’  5’-GCCAGGTCCTTCACTGTCTC-3’ |
| Nanog | 5’-CCCAAAGGCAAACAACCCACTTCT-3’  5’-AGCTGGGTGGAAGAGAACACAGTT-3’ |
| Sox2 | 5’-GCACATGAACGGCTGGAGCAACG-3’  5’-TGCTGCGAGTAGGACATGCTGTAGG-3’ |
| Oct4 | 5’-GTGGAGGAAGCTGACAACAA-3’  5’-ATTCTCCAGGTTGCCTCTCA-3’ |
| CD44 | 5’-CCGCTATGTCCAGAAAGGA-3’  5’-CTGTCTGTGCTGTCGGTGAT-3’ |
| CD133 | 5’-TGGATGCAGAACTTGACAACGT-3’  5’-ATACCTGCTACGACAGTCGTGGT-3’ |
| Bmi-1 | 5’-TGGAGAAGGAATGGTCCACTTC-3’  5’-GTGAGGAAACTGTGGATGAGGA-3’ |
| ABCC1 | 5’-TGCTGCACCAGTACTTCCACAT-3’  5’-CCCCAATGACAGCGGTCTT-3’ |
| ABCG2 | 5’-GCAGCTCTTCGGCTTGCA-3’  5’-CCCTGTTAATCCGTTCGTTTTT-3’ |
| ABCB1 | 5’- GCTCATCGTTTGTCTACAGTTCGT -3’  5’- ACAATGACTCCATCATCGAAACC -3’ |
| β-actin | 5’- AGAGCTACGAGCTGCCTGAC -3’  5’- AGCACTGTGTTGGCGTACAG -3’ |
