# Supplementary material for: PDK1- and PDK2-mediated metabolic reprogramming contributes to the TGFβ1-promoted stem-like properties in head and neck cancer
Source: Cancer Metab. 2022 Dec 6;10:23. doi: 10.1186/s40170-022-00300-0 (PMC9727917; doi:10.1186/s40170-022-00300-0)
Supplement: Supplementary file 2 — Additional file 2: Original, full-length blot images of Figure 2 B [file 40170_2022_300_MOESM2_ESM.docx]

Original, full-length blot images of Figure 2B.
